# Left ventricular myocardial strain responding to chronic pressure overload in patients with resistant hypertension evaluated by feature-tracking CMR

**DOI:** 10.1007/s00330-023-09595-z

**Published:** 2023-04-10

**Authors:** Hang Chen, Fabian J. Brunner, Cansu Özden, Ulrich O. Wenzel, Johannes T. Neumann, Jennifer Erley, Dennis Saering, Kai Muellerleile, Kai-Jonathan Maas, Bjoern P. Schoennagel, Ersin Cavus, Jan N. Schneider, Stefan Blankenberg, Andreas Koops, Gerhard Adam, Enver Tahir

**Affiliations:** 1 Department of Diagnostic and Interventional Radiology and Nuclear Medicine, University Medical Center Hamburg-Eppendorf, Hamburg, Germany; 2 Department of Cardiology, University Heart & Vascular Center Hamburg, University Medical Center Hamburg-Eppendorf, Martinistr. 52, 20246 Hamburg, Germany; 3 German Center for Cardiovascular Research (DZHK), Partner Site Hamburg/Kiel/Lübeck, Hamburg, Germany; 4 Department of Internal Medicine, Nephrology, University Medical Center Hamburg-Eppendorf, Hamburg, Germany; 5 Information Technology and Image Processing, University of Applied Sciences, Wedel, Germany; 6 Institute of Radiology and Interventional Therapy, Vivantes Auguste-Viktoria-Klinikum, Berlin, Germany

**Keywords:** Magnetic resonance imaging, Hypertension, Cardiac imaging techniques, Hypertrophy, left ventricular

## Abstract

**Objectives:**

The study aimed to investigate the alterations of myocardial deformation responding to long-standing pressure overload and the effects of focal myocardial fibrosis using feature-tracking cardiac magnetic resonance (FT-CMR) in patients with resistant hypertension (RH).

**Methods:**

Consecutive RH patients were prospectively recruited and underwent CMR at a single institution. FT-CMR analyses based on cine images were applied to measure left ventricular (LV) peak systolic global longitudinal (GLS), radial (GRS), and circumferential strain (GCS). Functional and morphological CMR variables, and late gadolinium enhancement (LGE) imaging were also obtained.

**Results:**

A total of 50 RH patients (63 ± 12 years, 32 men) and 18 normotensive controls (57 ± 8 years, 12 men) were studied. RH patients had a higher average systolic blood pressure than controls (166 ± 21 mmHg vs. 116 ± 8 mmHg, *p* < 0.001) with the intake of 5 ± 1 antihypertensive drugs. RH patients showed increased LV mass index (78 ± 15 g/m^2^ vs. 61 ± 9 g/m^2^, *p* < 0.001), decreased GLS (− 16 ± 3% vs. − 19 ± 2%, *p* = 0.001) and GRS (41 ± 12% vs. 48 ± 8%, *p* = 0.037), and GCS was reduced by trend (− 17 ± 4% vs. − 19 ± 4%, *p* = 0.078). Twenty-one (42%) RH patients demonstrated a LV focal myocardial fibrosis (LGE +). LGE + RH patients had higher LV mass index (85 ± 14 g/m^2^ vs. 73 ± 15 g/m^2^, *p* = 0.007) and attenuated GRS (37 ± 12% vs. 44 ± 12%, *p* = 0.048) compared to LGE − RH patients, whereas GLS (*p* = 0.146) and GCS (*p* = 0.961) were similar.

**Conclusion:**

Attenuation of LV GLS and GRS, and GCS decline by tendency, might be adaptative changes responding to chronic pressure overload. There is a high incidence of focal myocardial fibrosis in RH patients, which is associated with reduced LV GRS.

**Clinical relevance statement:**

Feature-tracking CMR-derived myocardial strain offers insights into the influence of long-standing pressure overload and of a myocardial fibrotic process on cardiac deformation in patients with resistant hypertension.

**Key Points:**

• *Variations of left ventricular strain are attributable to the degree of myocardial impairment in resistant hypertensive patients*.

• *Focal myocardial fibrosis of the left ventricle is associated with attenuated global radial strain*.

• *Feature-tracking CMR provides additional information on the attenuation of myocardial deformation responding to long-standing high blood pressure*.

**Supplementary Information:**

The online version contains supplementary material available at 10.1007/s00330-023-09595-z.

## Introduction

Among other cardiovascular risk factors hypertension remains a major cause of cardiovascular mortality worldwide [1]. Resistant hypertension (RH) is defined as above-goal elevated blood pressure despite the concurrent use of three or more different antihypertensive medications including a diuretic [2]. RH is associated with a higher risk of adverse cardiovascular events compared to controlled hypertension, and might be accompanied by extensive target organ damage, including left ventricular hypertrophy (LVH) [3, 4]. Myocardial fibrosis is a major determinant of hypertrophied myocardium and potentially associated with cardiovascular events, including heart failure and sudden death [5].

A recent work offers an overview of speckle-tracking echocardiography (STE) in assessing LV dysfunction in hypertension [6]. The explanation that attenuated longitudinal function and preserved circumferential and radial function are due to compensatory mechanisms has received reasonable attention, whereas longitudinal function is not always the earliest indicator in all circumstances, all three directions of function may decline in response to disease progress [6]. Although STE is the most available technique to quantify myocardial deformation, several weaknesses do exist. Reproducibility of acquisition planes is limited, which can influence particularly the evaluation of circumferential and radial strain [7]. The novel technique of feature-tracking cardiac magnetic resonance (FT-CMR), despite suffering from through-plane motion effects and having a lower spatial and temporal resolution than STE, has a better performance in measuring longitudinal, radial, and circumferential strain [7, 8]. Furthermore, the majority of the patient populations included in the previous echocardiographic literature had controlled mild to moderate hypertension [6].

Therefore, the main purpose of this study was to investigate the alterations of myocardial deformation responding to long-standing pressure overload and to elucidate the degree of myocardial impairment using FT-CMR. The secondary objective was to identify the potential effects of focal myocardial fibrosis in RH patients.

## Materials and methods

### Study population

The prospective study was approved by the local research ethics committee and complied with the Declaration of Helsinki. All participants gave written informed consent. This study recruited consecutive RH patients at a single institution and included 16 patients who were recruited in a previously publication [9]. The initial publication reported the effects of a renal denervation procedure on LV mass, myocardial strain, and diastolic function in RH patients [9].

The enrollment of criteria and diagnostic definitions have been detailed previously [9]. Briefly, RH patients were diagnosed according to the current guideline: blood pressure ≥ 140/90 mmHg despite the intake of at least 3 antihypertensive drugs in full dosages including a diuretic [2, 10]. The demographic and anthropometric characteristics were collected accordingly. Main exclusion criteria were as follows: (1) severe renal failure (estimated glomerular filtration rate [eGFR] < 30 mL/min/1.73 m^2^), (2) significant stenosis and prior stenting or dilatation of renal arteries, (3) myocardial infarction < 6 months before planned renal denervation, (4) diabetes mellitus type I, and (5) persisting atrial fibrillation [9]. All patients underwent an office and 24-h ambulant blood pressure monitoring (ABPM). In addition, 18 healthy individuals, who underwent CMR scans for this particular research purpose, were enrolled to serve as a control group and had no known cardiovascular or systematic diseases.

### CMR acquisition

CMR was performed on a 1.5-T scanner equipped with a 5-channel cardiac-phased array receiver coil (Achieva, Philips Medical Systems). Standard retrospectively gated, ECG-triggered steady-state free-precession cine images (25 phases per cardiac cycle) were acquired in short- and long-axis (2-, 3-, and 4-chamber) views using a breath-hold technique with the following typical parameters: acquired voxel size 1.98 × 1.80 × 6 mm^3^, reconstructed voxel size 1.36 × 1.36 × 6 mm^3^, gap 4 mm, 9–10 slices for full LV coverage, echo time = 1.67 ms, time to repetition = 3.34 ms, flip angle = 60°, parallel acquisition technique = SENSE [factor 2]). Ten minutes after a bolus injection of 0.2 mmol/kg gadoteric acid (Dotarem®, Guerbet) at a rate of 2.5 mL/s late gadolinium enhancement (LGE) images were acquired using an end-diastolic phase-sensitive inversion-recovery sequence in short-axis direction covering the entire heart and in 2-, 3-, and 4-chamber views.

### CMR data analysis

CMR images were post-processed independently and blindly using a commercially available software (CVi42, Circle Cardiovascular Imaging Inc.). CMR parameters are given as the mean of two investigators and are indexed to body surface area (BSA). For LV volume and mass evaluation, the endo- and epicardial contours were delineated in systole and diastole in a stack of short-axis cine slices covering the whole LV with inclusion of the papillary muscles as part of the LV volume [11]. For right ventricular (RV) volume evaluation, the endocardial contours were delineated in systole and diastole in a stack of short-axis cine slices covering the whole RV [11]. Left (LA) and right atrial (RA) volumes and LV ejection fraction (EF) were calculated based on the biplane area-length method [12], measurements excluded pulmonary veins and atrial appendage. LVH was defined as LV mass index > 81 g/m^2^ for men and > 61 g/m^2^ for women [13]. Focal myocardial fibrosis (LGE +) was identified and assessed visually using short- and long-axis LGE images. LV myocardial strain analysis was performed with cine images using the feature-tracking software (Segment, version 2.1.R.6108, Medviso), through computing interframe deformation fields using an endocardial tracking strategy based on non-rigid image registration [14, 15]. LV peak systolic global longitudinal (GLS), radial (GRS), and circumferential strain (GCS) were measured on the long-axis (2-, 3-, and 4-chamber) and three short-axis (apical, mid, and basal) slices by manual delineation of the endo- and epicardial contours at end-diastole. Endo- and epicardial contours were automatically propagated by the software throughout the cardiac cycle to calculate myocardial strain.

### Statistical analysis

All statistical analyses were performed using SPSS (version 28.0, IBM) and GraphPad Prism (version 9.2.0). All continuous data were checked for normality using the D’Agostino-Pearson omnibus normality test. Numerical variables are presented as the mean ± SD. Differences of continuous data between the groups were performed using the independent samples *t*-test or Wilcoxon signed rank-test as appropriate. Categorical data are presented as absolute numbers (percentage) and were compared using *χ*2 test or Fischer’s exact test as appropriate. Multivariate linear regression analyses were conducted to identify the independent associations of clinical and CMR-derived parameters with strain. *p* < 0.05 was regarded as statistically significant.

## Results

### Clinical characteristics

A total of 50 consecutive RH patients (63 ± 12 years, 32 men) and 18 normotensive controls (57 ± 8 years, 12 men) were eventually enrolled. A flowchart of the study is presented in Figure S1. Cardiovascular risk factors and antihypertensive medication of RH patients are detailed in Table 1. There were no statistical differences in gender distribution (*p* = 0.839) and age (*p* = 0.055) between RH patients and normotensive controls. RH patients had higher BSA (*p* = 0.004) and body mass index (BMI) (*p* < 0.001) than controls. Office systolic blood pressure (SBP) (166 ± 21 mmHg) and diastolic blood pressure (DBP) (91 ± 17 mmHg) were elevated in RH patients despite the intake of 5 ± 1 antihypertensive drugs. The mean of 24-h ABPM, SBP, and DBP of the patient group were 149 ± 18 mmHg and 84 ± 16 mmHg, respectively (Table 2).

**Table 1:** Clinical characteristics of RH patients

	RH patients (*n* = 50)
Risk factors	
Coronary artery disease, *n* (%)	13 (26)
Ischemic stroke, *n* (%)	4 (8)
Type 2 diabetes, *n* (%)	17 (34)
Smoker, *n* (%)	15 (30)
Hypercholesterolemia, *n* (%)	21 (42)
Antihypertensive medications	
Number of antihypertensive drugs, *n*	5 ± 1
Diuretics, *n* (%)	50 (100)
ACE inhibitors/ARBs, *n* (%)	41 (82)
Beta blockers, *n* (%)	39 (78)
Moxonidine or Clonidine, *n* (%)	28 (56)
Calcium channel blockers, *n* (%)	35 (70)
Aldosterone antagonists, *n* (%)	2 (4)
Alpha-adrenoreceptor antagonist, *n* (%)	8 (16)
Renin inhibitors, *n* (%)	9 (18)
Alpha-adrenoreceptor agonists, *n* (%)	2 (4)
Vasodilators, *n* (%)	4 (8)

Values are presented as mean ± SD for continuous data and *n* (%) for categorical data

*ACE* angiotensin-converting enzyme, *ARBs* angiotensin II receptor blockers, *RH* resistant hypertension

**Table 2:** Demographics, blood pressure, and CMR parameters in controls and RH patients as well as their subgroups stratified by the presence of LGE

	Controls (*n* = 18)	RH patients (*n* = 50)	*p* value^†^	LGE − RH patients (*n* = 29)	LGE + RH patients (*n* = 21)	*p* value^‡^
Demographics						
Age, years	57 ± 8	63 ± 12	0.055	62 ± 13	64 ± 10*	0.727
BSA, m^2^	1.90 ± 0.23	2.07 ± 0.21	**0.004**	2.01 ± 0.23	2.16 ± 0.15***	**0.017**
BMI, kg/m^2^	25 ± 4	31 ± 5	** < 0.001**	31 ± 6***	30 ± 4***	0.842
Male, *n* (%)	12 (67)	32 (64)	0.839	14 (48)	18 (86)	**0.007**
Blood pressure parameters						
Office SBP, mmHg	116 ± 8	166 ± 21	** < 0.001**	164 ± 39***	160 ± 18***	0.716
Office DBP, mmHg	77 ± 11	91 ± 17	**0.001**	88 ± 25	91 ± 16^**^	0.629
ABPM SBP, mmHg	–	149 ± 18	–	150 ± 18	148 ± 20	0.857
ABPM DBP, mmHg	–	84 ± 16	–	84 ± 16	84 ± 16	0.935
LV and LA CMR parameters						
LVEF, %	64 ± 7	62 ± 9	0.276	63 ± 8	59 ± 11	0.171
Heart rate, beats/min	67 ± 13	69 ± 11	0.499	69 ± 11	69 ± 11	0.879
LV mass index, g/m^2^	61 ± 9	78 ± 15	** < 0.001**	73 ± 15**	85 ± 14***	**0.007**
LVH all, *n* (%)	–	28 (56)	–	16 (55)	12 (57)	0.890
Male (mass > 81 g/m^2^), *n* (%)	–	14 (28)	–	5 (36)	9 (50)	0.419
Female (mass > 61 g/m^2^), *n* (%)	–	14 (28)	–	11 (73)	3 (100)	0.311
LVEDVi, mL/m^2^	75 ± 12	75 ± 16	0.856	72 ± 15	78 ± 16	0.155
LVESVi, mL/m^2^	27 ± 7	29 ± 11	0.388	27 ± 10	32 ± 12	0.119
LVSVi, mL/m^2^	48 ± 10	45 ± 9	0.227	45 ± 8	46 ± 11	0.578
LAEDVi, mL/m^2^	20 ± 6	24 ± 14	0.059	24 ± 14	25 ± 14	0.769
LAESVi, mL/m^2^	42 ± 11	43 ± 15	0.660	43 ± 13	44 ± 18	0.842
RV and RA CMR parameters						
RVEF, %	61 ± 6	63 ± 9	0.385	63 ± 8	62 ± 10	0.622
RVEDVi, mL/m^2^	74 ± 13	72 ± 13	0.460	72 ± 12	72 ± 15	0.889
RVESVi, mL/m^2^	29 ± 7	27 ± 8	0.313	26 ± 8	28 ± 9	0.575
RVSVi, mL/m^2^	46 ± 9	45 ± 10	0.837	45 ± 9	45 ± 11	0.785
RAEDVi, mL/m^2^	25 ± 10	23 ± 9	0.535	23 ± 8	23 ± 10	0.929
RAESVi, mL/m^2^	43 ± 14	39 ± 11	0.248	39 ± 9	39 ± 13	0.937
LV strain parameters						
LV GLS, %	− 19 ± 2	− 16 ± 3	**0.001**	− 16 ± 3*	− 15 ± 3***	0.146
LV GRS, %	48 ± 8	41 ± 12	**0.037**	44 ± 12	37 ± 12^**^	**0.048**
LV GCS, %	− 19 ± 4	− 17 ± 4	0.078	− 17 ± 4	− 17 ± 5	0.961
LGE pattern						
Ischemic, *n* (%)	–	7 (14)	–	–	7 (33)	–
Non-ischemic, *n* (%)	–	14 (28)	–	–	14 (67)	–

Values are presented as mean ± SD for continuous data and *n* (%) for categorical data

Values in **bold** denote significant differences between groups

^†^Comparison between RH patients and controls

^‡^Comparison between LGE − and LGE + RH patients

^*^p < 0.05, **p < 0.01, ***p < 0.001 for LGE − or LGE + RH patients vs. controls

*Abbreviations*: *ABPM*, ambulatory blood pressure monitoring; *BMI*, body mass index; *BSA*, body surface area; *DBP*, diastolic blood pressure; *GCS*, global circumferential strain; *GLS*, global longitudinal strain; *GRS*, global radial strain; *LA*, left atrial; *LAEDVi*, left atrial end-diastolic volume index; *LAESVi*; left atrial end-systolic volume index; *LGE*, late gadolinium enhancement; *LV*, left ventricular; *LVEF*, left ventricular ejection fraction; *LVEDVi*, left ventricular end-diastolic volume index; *LVESVi*, left ventricular end-systolic volume index; *LVH*, left ventricular hypertrophy; *LVSVi*, left ventricular stroke volume index; *RA*, right atrial; *RAEDVi*, right atrial end-diastolic volume index; *RAESVi*; right atrial end-systolic volume index; *RH*, resistant hypertension; *RV*, right ventricular; *RVEF*, right ventricular ejection fraction; *RVEDVi*, right ventricular end-diastolic volume index; *RVESVi*, right ventricular end-systolic volume index; *RVSVi*, right ventricular stroke volume index; *SBP*, systolic blood pressure

### CMR findings

**Fig. 1:**
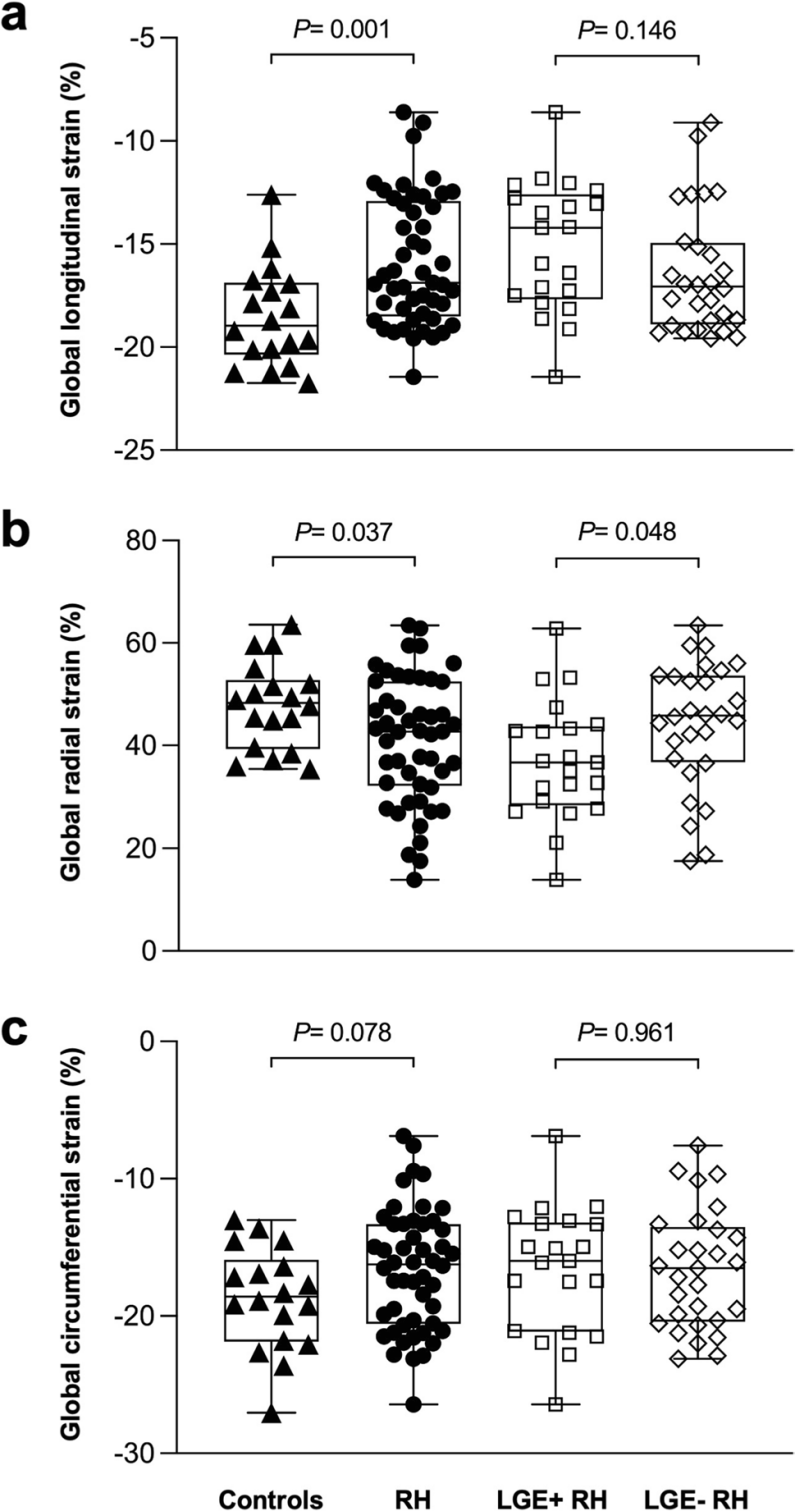
The comparisons of LV global longitudinal (**a**), radial (**b**), and circumferential (**c**) strain among RH patients with and without LGE and controls. LGE, late gadolinium enhancement; LV, left ventricular; RH, resistant hypertension

CMR findings of the study subjects are summarized in Table 2. RH patients showed similar LVEF (62 ± 9% vs. 64 ± 7%, *p* = 0.276) and LV/LA volumes, but markedly increased LV mass index (78 ± 15 g/m^2^ vs. 61 ± 9 g/m^2^, *p* < 0.001) compared to controls. In the patient group, 28 (56%) had LVH. No differences were observed between the groups regarding RV function and volumes. Feature-tracking analyses showed attenuated LV GLS (− 16 ± 3% vs. − 19 ± 2%, *p* = 0.001) and GRS (41 ± 12% vs. 48 ± 8%, *p* = 0.037) in RH patients. LV GCS had a downward tendency (− 17 ± 4% vs. − 19 ± 4%, *p* = 0.078) (Table 2 and Fig. 1).

Demographics, blood pressure, and CMR findings of LGE + and LGE − RH patients.

**Fig. 2:**
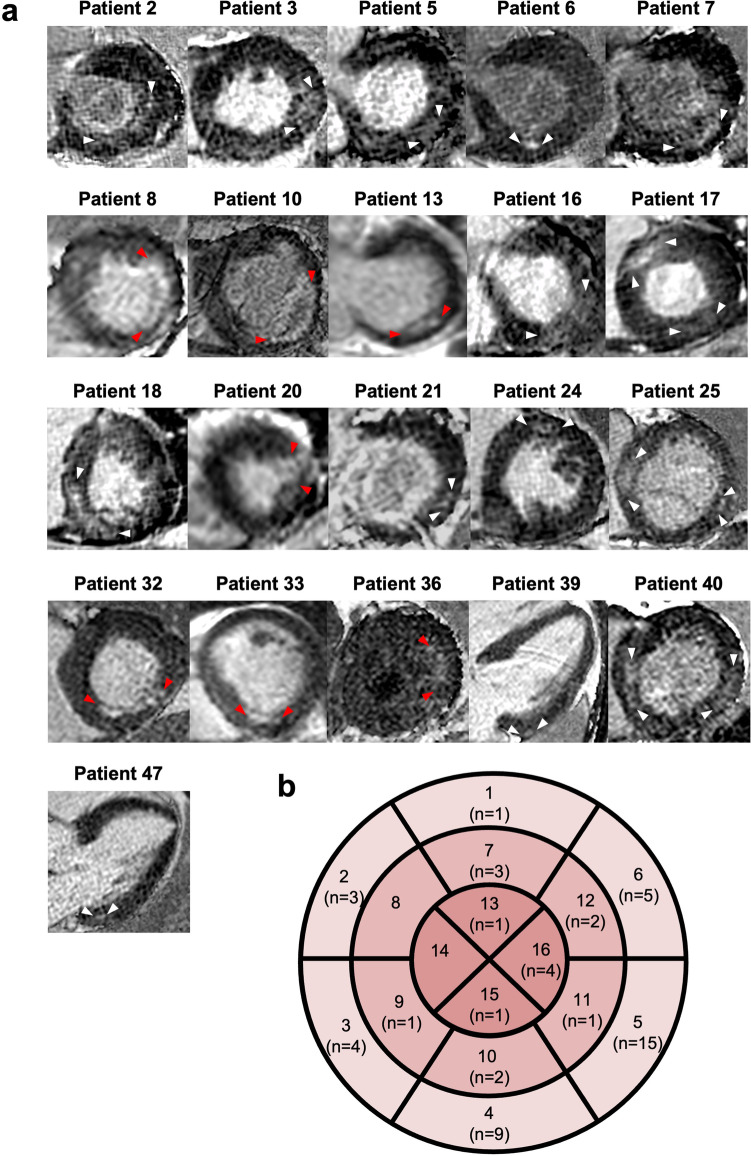
**a** LGE images depicting the focal myocardial fibrosis in LGE + RH patients. Short- and long-axis LGE images depicting an ischemic (red arrowheads) and non-ischemic (white arrowheads) pattern in 21 (42%) LGE + RH patients. **b** Schematic representation of fibrosis localization in LGE + RH patients. LGE areas were predominantly localized in the LV basal inferior (segment 4) and inferolateral (segment 5) segments, whereas midventricular anteroseptal (segment 8) and apical septal (segment 14) segments showed no focal myocardial fibrosis. LGE, late gadolinium enhancement; LV, left ventricular; RH, resistant hypertension

In 21 out of 50 (42%) RH patients, a focal myocardial fibrosis (LGE +) of the LV was detected. A total of 7 ischemic and 14 non-ischemic LGE patterns were visualized as shown in Fig. 2a. A schematic overview is given in Fig. 2b depicting the segmental distribution of focal myocardial fibrosis in LGE + RH patients. LGE areas were predominantly localized in the LV basal inferior and inferolateral segments, whereas midventricular anteroseptal and apical septal segments showed no focal myocardial fibrosis.

LGE − and LGE + RH patients had significantly higher BMI and office SBP than controls (all *p* < 0.001), increased LV mass index was found in both LGE − (*p* = 0.004) and LGE + (*p* < 0.001) RH patients. The two RH subgroups and controls had similar cardiac functional and anatomical parameters. Compared to normotensive controls, LV GLS was decreased in LGE + (− 15 ± 3% vs. − 19 ± 2%, *p* < 0.001) and LGE − RH patients (− 16 ± 3% vs. − 19 ± 2%, *p* = 0.015), GRS was decreased in LGE + RH (37 ± 12% vs. 48 ± 8%, *p* = 0.002), but not in LGE − RH patients (44 ± 12% vs. 48 ± 8%, *p* = 0.269) (Table 2). There were no statistical differences in LV GCS between controls and LGE − (*p* = 0.101) and LGE + (*p* = 0.127) RH patients, but a trend for attenuation.

There was a greater proportion of male patients in the LGE + RH group (*p* = 0.007) (Table 2). LGE + RH patients had higher BSA (2.16 ± 0.15 m^2^ vs. 2.01 ± 0.23 m^2^, *p* = 0.017) than LGE − RH patients. Age (*p* = 0.727) and BMI (*p* = 0.842) were similar. Office SBP (160 ± 18 mmHg vs. 164 ± 39 mmHg, *p* = 0.716) and DBP (91 ± 16 mmHg vs. 88 ± 25 mmHg, *p* = 0.629), ABPM SBP (148 ± 20 mmHg vs. 150 ± 18 mmHg, *p* = 0.857) and DBP (84 ± 16 mmHg vs. 84 ± 16 mmHg, *p* = 0.935) were similar. CMR revealed a higher LV mass index in LGE + RH patients with 85 ± 14 g/m^2^ than in LGE − RH patients with 73 ± 15 g/m^2^ (*p* = 0.007). Patients with LVH had a similar distribution between the LGE − and LGE + RH subgroups. No significant differences regarding cardiac function and volumes were observed (Table 2). Feature-tracking analyses showed that LGE + RH patients had attenuated LV GRS (37 ± 12% vs. 44 ± 12%, *p* = 0.048) compared to LGE − RH patients, whereas there were no differences in LV GLS (− 15 ± 3% vs. − 16 ± 3%, *p* = 0.146) and GCS (− 17 ± 5% vs. − 17 ± 4%, *p* = 0.961) (Fig. 1).

### Associations of clinical and CMR-derived parameters with strain

**Fig. 3:**
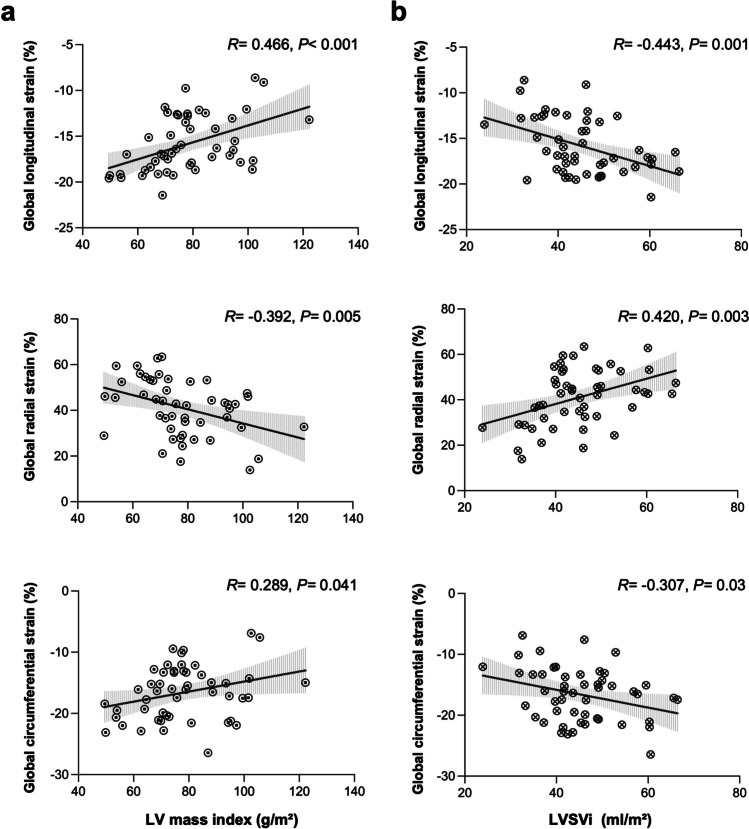
The associations of LV mass index (**a**) and LVSVi (**b**) with LV global deformation parameters in RH patients. The gray shade indicates the 95% confidence interval. Increase in LV mass index and decrease in LVSVi were associated with decrease in longitudinal, radial, and circumferential strain. LV, left ventricular; LVSVi, left ventricular stroke volume index; RH, resistant hypertension

Univariate regression analyses showed that LV stroke volume index (LVSVi) and LV mass index in RH patients were associated with LV GLS (*R* =  − 0.443, *p* = 0.001 and *R* = 0.466, *p* < 0.001, respectively), GRS (*R* = 0.420, *p* = 0.003 and *R* =  − 0.392, *p* = 0.005, respectively), and GCS (*R* =  − 0.307, *p* = 0.03 and *R* = 0.289, *p* = 0.041, respectively) (Fig. 3). After adjustment for age, gender, BMI, and BP, multivariate regression analyses demonstrated that LV end-systolic volume index (LVESVi), LVSVi, and LV mass index were independently associated with LV GLS (*β* = 0.301, *p* = 0.002; *β* =  − 0.689, *p* < 0.001 and *β* = 0.558, *p* < 0.001, respectively; model *R*^*2*^ = 0.713) and GRS (*β* =  − 0.447; *β* = 0.616 and *β* =  − 0.379, respectively, all* p* < 0.001; model *R*^*2*^ = 0.685). LVESVi and LVSVi were independently associated with LV GCS (*β* = 0.711,* p* < 0.001; *β* =  − 0.413, *p* < 0.001, respectively; model *R*^*2*^ = 0.588) (Table 3). Multivariate regression analyses in normotensive controls showed that considering the covariates of age, gender, BMI, and BP, LVSVi was independently associated with LV GLS (*β* =  − 0.521, *p* = 0.027, model *R*^*2*^ = 0.271), LVESVi was independently associated with LV GRS (*β* =  − 0.675, *p* = 0.002, model *R*^*2*^ = 0.456), and LVESVi and LVSVi were independently associated with LV GCS (*β* = 0.722 and *β* =  − 0.615, all *p* < 0.001; model *R*^*2*^ = 0.746) (Table 4).

**Table 3:** Univariate and multivariate linear regression analysis of clinical factors and CMR parameters on LV deformation in RH patients

	LV GLS		LV GRS		LV GCS	
	Univariate	Multivariate	Univariate	Multivariate	Univariate	Multivariate
	*R*	*β*	*R*	*β*	*R*	*β*
Clinical factors						
Age (years)	0.116		− 0.194	− 0.183*	− 0.128	
BSA (m^2^)	0.089		− 0.044		0.116	
BMI (kg/m^2^)	0.121		0.008		0.130	
Male	− 0.309*		0.224		− 0.087	
Office SBP (mmHg)	0.251		− 0.113		0.017	
Office DBP (mmHg)	0.374**		− 0.380**		0.070*	
CMR parameters						
Heart rate (beats/min)	0.337*		− 0.374**		0.364**	
LVEDVi (mL/m^2^)	0.089		− 0.141		0.286*	
LVESVi (mL/m^2^)	0.489***	0.301**	− 0.544***	− 0.447***	0.649***	0.711***
LVSVi (mL/m^2^)	− 0.443**	− 0.689***	0.420**	0.616***	− 0.307*	− 0.413***
LV mass index (g/m^2^)	0.466***	0.558***	− 0.392**	− 0.379***	0.289*	
RVEDVi (mL/m^2^)	− 0.133		0.074		0.114	
RVESVi (mL/m^2^)	0.278		− 0.339*		0.337*	
RVSVi (mL/m^2^)	− 0.404**		0.375**		− 0.118	
LAEDVi (mL/m^2^)	0.153		− 0.169		0.126	
LAESVi (mL/m^2^)	− 0.139		0.046		− 0.005	
RAEDVi (mL/m^2^)	0.264		− 0.338*		0.317*	
RAESVi (mL/m^2^)	− 0.034		− 0.106		0.138	
*R*^*2*^		0.713		0.685		0.588

Variates with *p* < 0.05 in the univariate analysis as well as age, gender, BMI, SBP, and DBP were included in the multivariate analysis. *β* is the standardized regression coefficient of stepwise multivariate linear regression analysis

^*^p < 0.05; **p < 0.01; ***p < 0.001

Abbreviations as in Tables 1 and 2

**Table 4:** Univariate and multivariate linear regression analysis of clinical factors and CMR parameters on LV deformation in the control group

	LV GLS		LV GRS		LV GCS	
	Univariate	Multivariate	Univariate	Multivariate	Univariate	Multivariate
	*R*	*β*	*R*	*β*	*R*	*β*
Clinical factors						
Age (years)	− 0.045		0.266		− 0.018	0.395*
BSA (m^2^)	− 0.317		0.169		0.002	
BMI (kg/m^2^)	− 0.047		0.204		0.053	
Male	0.136		0.074		0.182	
Office SBP (mmHg)	0.138		0.263		− 0.047	
Office DBP (mmHg)	− 0.172		0.114		− 0.282	
CMR parameters						
Heart rate (beats/min)	0.251		− 0.136		0.038	
LVEDVi (mL/m^2^)	− 0.236		− 0.203		− 0.125	
LVESVi (mL/m^2^)	0.290		− 0.675**	− 0.675**	0.543*	0.722***
LVSVi (mL/m^2^)	− 0.521*	− 0.521*	0.272		− 0.587*	− 0.615***
LV mass index (g/m^2^)	− 0.096		− 0.045		− 0.415	
RVEDVi (mL/m^2^)	− 0.064		− 0.251		− 0.287	
RVESVi (mL/m^2^)	0.120		− 0.336		0.187	
RVSVi (mL/m^2^)	− 0.175		− 0.084		− 0.567*	
LAEDVi (mL/m^2^)	− 0.007		− 0.028		0.464	
LAESVi (mL/m^2^)	− 0.377		0.083		0.149	
RAEDVi (mL/m^2^)	− 0.109		0.108		0.146	
RAESVi (mL/m^2^)	− 0.222		0.004		0.030	
*R*^*2*^		0.271		0.456		0.746

Variates with *p* < 0.05 in the univariate analysis as well as age, gender, BMI, SBP, and DBP were adjusted in the multivariate analysis. *β *is the standardized regression coefficient of stepwise multivariate linear regression analysis

^*^p < 0.05; **p < 0.01; ***p < 0.001

Abbreviations as in Tables 1 and 2

## Discussion

This study analyzed cardiac morphology and function in RH patients compared to normotensive controls using CMR. The novel method of FT-CMR was used to determine LV global peak systolic strain and LGE imaging was used to investigate the influence of focal myocardial fibrosis on LV myocardial deformation. The main findings are (1) RH patients had significantly higher LV mass index and attenuated LV GLS and GRS in comparison to normotensive controls, whereas GCS was attenuated by trend; (2) 21 RH patients (42%) demonstrated a focal myocardial fibrosis (LGE +), predominantly localized in the basal inferior and inferolateral LV segments; (3) in the subgroup analysis, LGE + RH patients had a markedly reduced LV GLS compared to controls, attenuated GRS compared to LGE − RH patients and controls, and GCS was also attenuated by trend; and (4) LV mass index and stroke volume index were associated with multidirectional strain in RH patients.

### Long-standing pressure overload causing strain alterations

In this study, a decrease of LV GLS and GRS was observed in RH patients compared to normotensive controls, GCS did not differ statistically, but showed a decreasing trend. LV strain is sensitive to and influenced by afterload alteration [16], and its altering patterns are determined by the fiber structure of the myocardium and its interaction with local wall stress [17]. Longitudinal strain represents the contraction of the subendocardial fibers, while circumferential shortening reflects the contraction of the subepicardial fibers, and both contribute to radial thickening [8]. LV subendocardial fibers are more vulnerable to increased wall stress, ischemia, and microvascular dysfunction, and thus longitudinal strain is prone to impairment at an early phase of hypertension even before hypertrophy has occurred [18–20] and is a sensitive marker for subclinical LV dysfunction [17, 21].

The alterations in radial and circumferential strain are more complex compared to longitudinal strain, especially with the progression of the given disease and the presence of LVH. Imbalzano et al detected reduced longitudinal strain by STE in hypertensive patients both with and without LVH, and those with LVH had reduced radial and increased circumferential strain [18]. Wang et al showed a reduction in all three strain components in patients with systolic heart failure, while patients with diastolic heart failure and preserved LVEF had reduced longitudinal and radial strain, but circumferential strain was preserved [21]. In the current cohort, longitudinal and radial strain was decreased and circumferential strain was preserved in analogy, and more than half (56%) of the patients had LVH due to persistent high-pressure overload. The anatomic differences of myocardial fibers may explain the potential robustness of circumferential strain in terms of clinically significant LV dysfunction [22]. Thus, different stages of hypertensive heart disease seem to be associated with different longitudinal, radial, and circumferential strain response, which may provide a possible explanation for the above discrepancy.

Of note, in the current study, there was a borderline difference of age between controls and RH patients; age-dependency may contribute to the compensation of age-related LV stiffness by radial strain [23]. However, a multivariate regression analysis after adjustment for age showed that RH remained independently associated with LV GLS and GRS, but not with GCS (Table S1).

Taken together, our results support the notion that the attenuation of longitudinal and radial strain in RH patients might constitute a LV adaptation as a response to a long-standing pressure overload. The tendential decrease of circumferential strain might be explained with the subepicardial layer being affected to a lesser degree in this RH patient cohort.

### Prevalence of LGE in RH

Recently, an observational study reported that 145 (18%) of 786 patients with essential hypertension had non-ischemic LGE; they were more likely to be men and had greater LV mass and decreased strain [24]. Also, Wang et al detected 29.9% LGE + in their hypertension group [25]. In contrast, our cohort showed a higher prevalence of LGE (42%) with a predominantly non-ischemic pattern, suggesting that RH might be associated with a higher prevalence of LGE than controlled hypertension.

Myocardial fibrosis is a common end point of many cellular and noncellular pathological processes in hypertension; the severity and duration of hypertension might be responsible for the development of cardiac remodeling [26, 27]. In our study, LGE + RH patients had higher LV mass index; increased LV mass in remodeling is due to expanded extracellular interstitium and myocardial cell volume [28]. In the presence of an expanded interstitium, focal replacement fibrosis (non-ischemic LGE) is regarded as a result from the progression of interstitial fibrosis [24]. Increased collagen deposition in the extracellular interstitium induces stiffness and reduction of end-diastolic myofiber length, consequently inducing weakened contraction [29].

### Differences in strain between LGE + and LGE − RH patients

Our results showed that reduction in longitudinal strain was observed in both LGE* − *and LGE + RH patients, compatible with an early decrease of longitudinal systolic function. However, while LGE* − *RH patients showed similar radial strain compared to controls, a worsening radial strain emerged in LGE + RH patients.

Generally, radial strain has been shown to have large ranges between studies and the variability of segmental strain remains rather high [8]. Nevertheless, radial strain can help to distinguish cardiac sarcoidosis from dilated cardiomyopathy [30], can predict clinical outcome in hypertrophic cardiomyopathy [31], and is more predictive for scar (defined with LGE) transmurality than longitudinal strain [32]. In fact, the underlying mechanisms responsible for worsening radial strain have not been completely defined yet. Radial strain represents the global myocardial function in the radial direction, which is influenced by the deformation of all myocardial layers. Thus, it seems reasonable to assume that once focal myocardial fibrosis visualizable by LGE has occurred it might contribute to the reduction of LV radial strain.

Earlier echocardiographic studies have investigated the effects of myocardial fibrosis on LV deformation through identifying the association of plasma markers of myocardial fibrosis with strain alterations. Kang et al found increasing tissue inhibitor of matrix metalloproteinase (TIMP)-1 in hypertensive patients with normal LVEF correlated with attenuation of longitudinal strain, whereas circumferential and radial strain were not attenuated [19]. Poulsen et al showed that hypertensive patients had decreased longitudinal strain and increased amino-terminal propeptide of procollagen type III, accompanied by an inverse correlation of the two parameters [20]. Plasma markers emerge in an early stage of a myocardial fibrotic process in mild to moderate hypertensive patients and indirectly reflect myocardial fibrosis, and may lack specificity in the case of concomitant fibrotic diseases (e.g., cardiac fibrosis combined with liver or kidney fibrosis) [33]. However, the patients in the current study rather suffered a late fibrotic process due to long-standing arterial hypertension. LGE-CMR is a visual approach to directly display focal myocardial fibrosis [34]. Identifying the strain differences in RH patients with and without focal myocardial fibrosis might provide data on the extent of myocardial layer impairment and offer insights into the influence of a long-standing pressure overload and a myocardial fibrotic process on cardiac deformation.

### Limitations

The sample size in our study was small, which may have had an influence on the power to identify differences between study groups. However, all participants were recruited consecutively and prospectively according to the stringent selection criteria; future studies with larger populations are warranted to corroborate the consistency and reproducibility of our preliminary findings. Second, although some risk factors had been adjusted for multivariate regression analyses, several potential confounders, such as the dosages of antihypertensive drugs and sodium intake, may have an additional effect. Age-matching of controls and RH patients was not precise, but a multivariate analysis did not alter the results after adjustment for age. Another limitation is the lack of detailed information about the duration of hypertension. Nevertheless, all recruited patients were classified as RH according to the ESC guidelines and extensive diagnostics had been previously performed at our tertiary university medical center excluding secondary causes of hypertension. It can be assumed that hypertension has been developing over a long period of time during the aging process in the vast majority of the current elder cohort. Further, FT-CMR is performed mainly based on a block-matching algorithm, which requires a careful tuning of the search region and solving for displacements between short-distance regions [7]. Thus, radial strain being calculated over smaller regions between endo- and epicardium is less reliable than longitudinal and circumferential strain [7].

## Conclusions

Our study revealed that attenuation of LV global longitudinal and radial strain as well as the tendency of circumferential strain attenuation might be consecutive adaptations responding to long-standing pressure overload in RH patients, and global circumferential strain attenuation only by tendency might be attributable to a still partially preserved subepicardial layer. Further, focal myocardial fibrosis has a high incidence in RH patients, presents primarily with a non-ischemic LGE pattern predominantly localized in the basal inferior and inferolateral LV segments, and is associated with reduced global radial strain. Therefore, FT-CMR-derived myocardial strain offers insights into the influence of long-standing pressure overload and of a myocardial fibrotic process on cardiac deformation in RH.

## Supplementary Information

Below is the link to the electronic supplementary material.

Supplementary file1 (PDF 168 KB)

## Abbreviations

ABPM: Ambulant blood pressure monitoring
BMI: Body mass index
BSA: Body surface area
CMR: Cardiac magnetic resonance
DBP: Diastolic blood pressure
EDVi: End-diastolic volume index
EF: Ejection fraction
ESVi: End-systolic volume index
FT-CMR: Feature-tracking cardiac magnetic resonance
GCS: Global circumferential strain
GLS: Global longitudinal strain
GRS: Global radial strain
LA: Left atrial
LGE: Late gadolinium enhancement
LV: Left ventricular
LVH: Left ventricular hypertrophy
RA: Right atrial
RH: Resistant hypertension
RV: Right ventricular
SBP: Systolic blood pressure
SSFP: Steady-state free-precession
STE: Speckle-tracking echocardiography
SVi: Stroke volume index

## Declarations

### Guarantor

The scientific guarantor of this publication is Enver Tahir, MD.

### Statistics and biometry

No complex statistical methods were necessary for this paper.

### Previous reports of the study cohort

The study population included 16 RH patients, who were included in a previously publication. The initial publication reported on the effects of a renal denervation procedure in RH patients. In brief, changes in LV mass, myocardial strain, and diastolic function were assessed before and on a 12-month follow-up after renal denervation.

Tahir E, Koops A, Warncke ML et al (2019) Effect of renal denervation procedure on left ventricular mass, myocardial strain and diastolic function by CMR on a 12-month follow-up. Jpn J Radiol 37:642-650.

### Methodology

• prospective

• observational

• performed at one institution
