# Supplementary material for: Left ventricular myocardial strain responding to chronic pressure overload in patients with resistant hypertension evaluated by feature-tracking CMR
Source: Eur Radiol. 2023 Apr 10;33(9):6278–89. doi: 10.1007/s00330-023-09595-z (PMC10415476; doi:10.1007/s00330-023-09595-z)

**Table S1:** Age-adjusted association of RH with LV deformation.

| LV strain parameters | Unstandardized coefficient† | 95% CI | *P* value |
|---|---|---|---|
| LV GLS, % | 2.684 | 1.071, 4.297 | **0.001** |
| LV GRS, % | -6.695 | -12.987, -0.402 | **0.037** |
| LV GCS, % | 2.333 | -0.060, 4.726 | 0.056 |

†Unstandardized coefficient of RH adjusted by age.

Values in **bold** denote statistical significance.

**Abbreviations:** CI, confidence interval; GCS, global circumferential strain; GLS, global longitudinal strain; GRS, global radial strain; LV, left ventricular; RH, resistant hypertension.

**Figure S1:** Flow chart of the study.

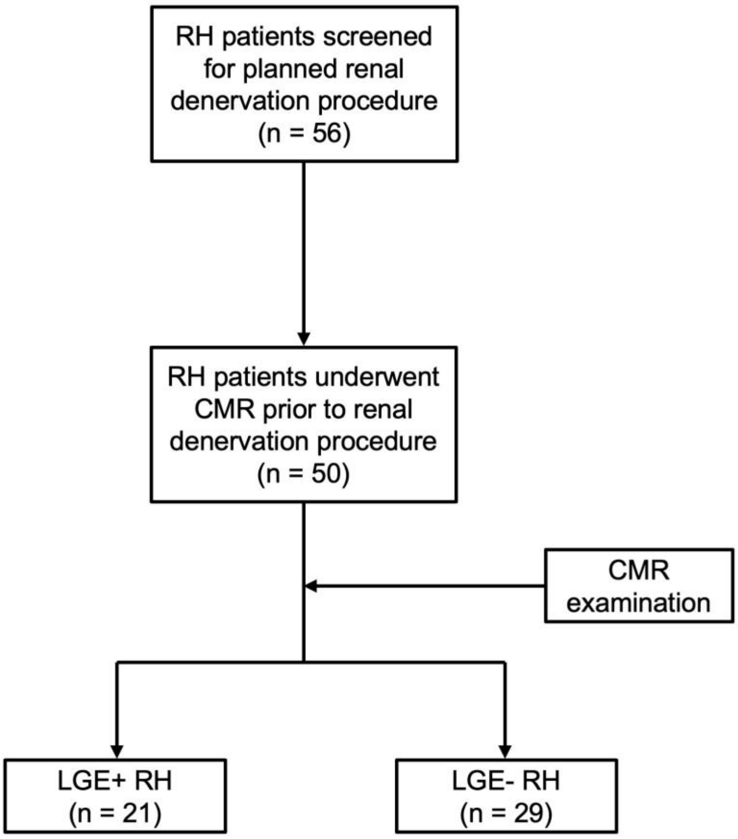

Supplement: Supplementary file 1 — Supplementary file1 (PDF 168 KB) [file 330_2023_9595_MOESM1_ESM.pdf]
